# Colloidal inorganic nano- and microparticles for passive daytime radiative cooling

**DOI:** 10.1186/s40580-023-00365-7

**Published:** 2023-04-18

**Authors:** Ho Young Woo, Yoonjoo Choi, Hyesun Chung, Da Won Lee, Taejong Paik

**Affiliations:** School of Integrative Engineering, Chung-Ang University, Seoul, 06974 Republic of Korea

**Keywords:** Radiative cooling, Nanoparticle, Microparticle, Colloid, Multifunctionality, Thermal management

## Abstract

Compared to traditional cooling systems, radiative cooling (RC) is a promising cooling strategy in terms of reducing energy consumption enormously and avoiding severe environmental issues. Radiative cooling materials (RCMs) reduce the temperature of objects without using an external energy supply by dissipating thermal energy via infrared (IR) radiation into the cold outer space through the atmospheric window. Therefore, RC has a great potential for various applications, such as energy-saving buildings, vehicles, water harvesting, solar cells, and personal thermal management. Herein, we review the recent progress in the applications of inorganic nanoparticles (NPs) and microparticles (MPs) as RCMs and provide insights for further development of RC technology. Particle-based RCMs have tremendous potential owing to the ease of engineering their optical and physical properties, as well as processibility for facile, inexpensive, and large area deposition. The optical and physical properties of inorganic NPs and MPs can be tuned easily by changing their size, shape, composition, and crystals structures. This feature allows particle-based RCMs to fulfill requirements pertaining to passive daytime radiative cooling (PDRC), which requires high reflectivity in the solar spectrum and high emissivity within the atmospheric window. By adjusting the structures and compositions of colloidal inorganic particles, they can be utilized to design a thermal radiator with a selective emission spectrum at wavelengths of 8–13 μm, which is preferable for PDRC. In addition, colloidal particles can exhibit high reflectivity in the solar spectrum through Mie-scattering, which can be further engineered by modifying the compositions and structures of colloidal particles. Recent advances in PDRC that utilize inorganic NPs and MPs are summarized and discussed together with various materials, structural designs, and optical properties. Subsequently, we discuss the integration of functional NPs to achieve functional RCMs. We describe various approaches to the design of colored RCMs including structural colors, plasmonics, and luminescent wavelength conversion. In addition, we further describe experimental approaches to realize self-adaptive RC by incorporating phase-change materials and to fabricate multifunctional RC devices by using a combination of functional NPs and MPs.

**Graphical Abstract:**

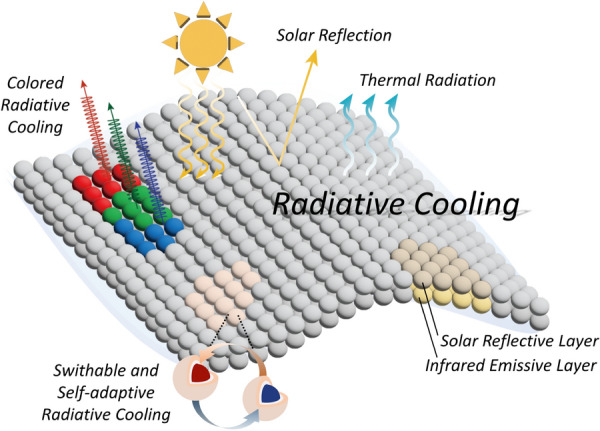

**Supplementary Information:**

The online version contains supplementary material available at 10.1186/s40580-023-00365-7.

## Introduction

Traditional cooling systems that use electricity require a considerable amount of energy, leading to multiple problems such as excessive CO_2_ gas emissions, ozone depletion, energy shortage, and greenhouse effects [1–3]. Therefore, there is a tremendous demand for new energy-saving, maintenance-free, inexpensive, and environmentally friendly approaches that can replace the traditional electricity-based cooling systems. Radiative cooling (RC) has recently attracted significant attention as a promising temperature-lowering strategy that does not require external energy for cooling [4–8]. RC materials (RCMs) have great potential for use in various applications, such as energy-saving buildings [9–13], vehicles [14–16], water harvesting [17, 18], solar cells [19, 20], and personal thermal management [21–23]. RCMs spontaneously reduce the temperature of a system by releasing thermal energy into the cold sink of outer space through infrared (IR) radiation [6, 24, 25]. Molecules in the atmosphere (H_2_O, N_2_, O_2_, O_3_, CO_2_) absorb, emit, reflect, and scatter terrestrial thermal radiation, and these interactions impede the emission of thermal radiation from the Earth’s surface into space [24]. However, in the 8–13 μm wavelength range, which is called the atmospheric transparency window, thermal energy does not interact with the atmosphere, which allows most thermal radiation to pass through the atmosphere. Thus, thermal energy can be efficiently released into space resulting in the cooling of objects. RCMs require high radiative thermal emissivity in the atmospheric window, that is, in the wavelength range of 8–13 μm, to maximize radiative heat loss.

According to Kirchhoff’s law of thermal radiation, the angular spectral absorption and emission are equal at thermal equilibrium. Therefore, when the ambient temperature is higher than that of RCMs, the RCMs re-absorb thermal emission from the atmosphere, which reduce its cooling capability [6]. Hence, for sub-ambient RC, RCMs should possess selective emissivity within the atmospheric window to reduce the absorption of incoming radiative heat from the atmosphere. By contrast, in the daytime under direct sunlight, solar heating must be considered. The global AM1.5 solar spectrum has an irradiance of approximately 1000 W/m^2^, and the absorption of even a small amount of solar radiation could exceed and offset the thermal radiation from the radiator, thereby reducing the cooling effect of RCMs [5]. Therefore, RCMs must possess high reflectivity in the solar spectrum to minimize the absorption of solar illumination and achieve sub-ambient daytime cooling. IR emission and solar absorption are significant factors in the design of RCMs. The fundamental principles of RC provide the concepts and requirements for efficient RC designs by using the following equation. Let us consider a RCMs at a temperature T with spectral and angular emissivity of $\varepsilon \left(\lambda ,\theta \right)$, respectively, that is exposed to direct sunlight. Its net cooling power (${P}_{net}$) can expressed as follows [26].

1 $$P_{net} \left( T \right) = P_{rad} \left( T \right) - P_{atm} \left( {T_{atm} } \right) - P_{sun} - P_{non - rad,}$$

where

2 $$P_{rad} \left( T \right) = \mathop \smallint \limits_{0}^{2\pi } \mathop \smallint \limits_{0}^{{\frac{\pi }{2}}} \mathop \smallint \limits_{0}^{\infty } I_{BB} \left( {T,\lambda } \right)\varepsilon \left( {\lambda ,\theta } \right)cos\theta sin\theta d\lambda d\theta d\varphi$$

is the thermal radiative power from the surface of the RCMs. Here, ${I}_{BB}=\left(2h{c}^{2}/{\lambda }^{5}\right)/\left[{e}^{hc/\lambda {k}_{B}T}-1\right]$ is the spectral radiance of a blackbody at temperature T and wavelength $\lambda$ according to Planck’s law. $h$, $c$, and, ${k}_{B}$ are Planck’s constant, the speed of light, and Boltzmann’s constant, respectively. The absorbed atmospheric radiation power on the RCM surface is

3 $$P_{atm} \left( {T_{atm} } \right) = \mathop \smallint \limits_{0}^{2\pi } \mathop \smallint \limits_{0}^{{\frac{\pi }{2}}} \mathop \smallint \limits_{0}^{\infty } I_{BB} \left( {T_{atm} ,\lambda } \right)\varepsilon \left( {\lambda ,\theta } \right)\varepsilon_{atm} \left( {\lambda ,\theta } \right)cos\theta sin\theta d\lambda d\theta d\varphi$$

The spectral and angular emissivity of the atmosphere is ${\varepsilon }_{atm}\left(\lambda ,\theta \right)=1-{t\left(\lambda \right)}^{1/cos\left(\theta \right)}$, where $t\left(\lambda \right)$ is the atmospheric IR transmittance along the zenith direction [4].

4 $$P_{sun} = \mathop \smallint \limits_{0}^{\infty } I_{solar} \left( \lambda \right)\varepsilon \left( {\lambda ,\theta } \right)d\lambda$$

is the radiative power absorbed by the RCMs from solar radiation, where ${I}_{solar}$ is the AM1.5 solar intensity with an irradiance of approximately 1000 W/m^2^ [27]. Solar irradiation can be absorbed and converted into heat. The absorption of a small amount power in the solar spectrum can easily reduce the cooling efficiency of RCMs, For this reason, solar absorptivity must be considered when estimating the RC efficiency of daytime RC. Moreover, non-radiative heat transfer, conduction, and convection between an RCM and its surroundings should be considered to evaluate the RC performance, as follows [4, 7]:

5 $$P_{non - rad} = h_{c} \left( {T_{atm} - T} \right)$$

where ${h}_{c}$ is the heat transfer coefficient stemming from the conductive and convective heat exchange of RCMs with the surroundings. Conduction heat transfer is affected by the surroundings, and convection heat transfer depends on the wind speed over RCMs. The cooling power of RCMs in non-radiative heat transfer decreases in the absence of insulation. Therefore, for sub-ambient cooling, RCMs should be designed to minimize the effect of non-radiative heat transfer, and, generally, RCMs should be covered with insulators possessing high IR transmittance to reduce their non-radiative heat gain [5]. Solar absorption and IR emission are important factors for improving the cooling power of RCMs. Therefore, RCMs should possess high reflectivity in the 0.3–2.5 μm wavelength range to minimize daytime solar absorption. Moreover, selective high emissivity within the atmospheric transparency window of 8–13 μm is required to ensure that RCMs can dissipate thermal energy through IR radiation into outer space.

In the early stage of research on RC in the twentieth century, many researchers focused on the development of RCMs with high thermal emissivity in the atmospheric window by using polymer films [28–30], pigmented paints [31–33], inorganic films [34–38], and gaseous materials [39–41]. High IR emissivity facilitates effective RC at nighttime. However, for passive daytime radiative cooling (PDRC), as described previously, radiative heat emission from RCMs should be greater than the absorption of incoming solar energy. Raman et al. experimentally demonstrated high solar reflection and thermal emission for PDRC materials with multilayer photonic structures [4]. These photonic structures comprised seven alternating layers of hafnia (HfO_2_) and silica (SiO_2_) on the reflective silver substrates to achieve 97% of solar reflectivity, while exhibiting strong selective emission within the atmospheric window, and a temperature reduction of 4.9 ℃ relative to the ambient temperature under direct sunlight. Since then, the development of PDRC materials has attracted significant interest from researchers, and various types of RCMs with high solar reflectivity and high atmospheric emissivity have been reported, including nanophotonic structures [4, 26, 42, 43], particle or polymeric coatings [44–46], porous polymers [47–49], and nanoparticle-polymer composites [50–54]. Nanophotonic structures reflect sunlight efficiently and emit IR radiation strongly. However, their fabrication requires a vacuum-chamber-based process, which limits the development of a large-area and inexpensive process for commercial application. Polymer-based PDRC materials are suitable in terms of cost and processibility, although they have drawbacks such as polymer degradation under ultraviolet (UV) irradiation. These PDRC structures often require reflective metal substrates to achieve high solar reflectance under direct solar radiation. However, the inherent solar absorption characteristics of metal reflectors and oxidation of the metal layer reduce solar reflectance, and thus, RC performance.

Meanwhile, colloidal particle-based PDRC materials have tremendous potentials owing to their superior optical and physical properties, as well as processibility. Various types of nanoparticles (NPs) and microparticles (MPs) including metal, semiconductors, and insulators can be synthesized by tailoring their sizes, shapes, and compositions and such particles are commercially available for use in pigments and paints. In addition, colloidal inorganic NPs and MPs can be easily deposited on various types of substrates through large-area, solution-processed fabrication methods such as spray-coating and roll-to-roll coating. The properties of inorganic NPs and MPs can be tuned readily by changing their structures and compositions [55]. Recently, many researchers have attempted to design particle-based PDRC materials for selective thermal emitters in the atmospheric window and for use in RC paints [51, 54, 56–58], fabrics [23, 50, 59–61], and composite films [16, 52, 62]. In addition to IR emission, incident solar light can be scattered effectively by particles of sizes comparable to solar wavelength through Mie-scattering. In this manner, these particles can serve as efficient solar reflectors in PDRC materials [63]. Because colloid-based PDRC materials that utilize Mie-scattering do not use reflective metal layers, solar absorption from metallic substrates can be reduced, leading to a reduction in fabrication cost and enhancement of RC performance. Therefore, particle-based radiators are one of the promising PDRC materials.

In this review, we introduce recent advances in and strengths of PDRC materials that employ inorganic NPs and MPs, especially in terms of their application as solar reflectors, thermal emitters, aesthetic colorants, and multifunctionality materials. We first summarize the materials, structural designs, optical properties of various RCMs that employ inorganic NPs and MPs to achieve high thermal emittance in the atmospheric window and high solar reflectance for efficient PDRC. Inorganic NPs and MPs possess unique physical and chemical properties that are different from those of their bulk counterparts, and as a result, they have attracted significant from researchers in terms of their use in RCMs. For example, in the spectral regime of the optical phonon mode, resonant excitation, also called surface phonon polariton is induced on the particle surface when photons are absorbed while interacting with phonons or vibrons [54, 64]. Optical phonon absorption in ionic dielectrics can be tailored by changing on the sizes, chemical bonds, and compositions of the particles. By designing the structure of NPs and MPs such that they exhibit selective absorption in the wavelength range of 8–13 μm, high emissivity can be achieved in the atmospheric window. In addition, solar reflectance increases when the particle size is comparable to the wavelength of the incident light, which minimizes the absorption of solar energy. These novel RCMs and strategies are discussed and summarized in terms of the features and characteristics of inorganic NPs and MPs. Moreover, we introduce recent advances in colored RCMs that exploit various aspects of particles such as structural color and plasmonic properties, to minimize solar absorption through selective narrow absorption in the visible region and luminescent wavelength converters. Furthermore, we highlight the development of adaptive RC and multifunctional RC by introducing functional NPs and MPs or phase-change materials into RCMs. This review presents the general concepts of particle-based RCMs and provides key insights for further development of RC technology.

## Inorganic nano- and microparticles for RCMs

**Fig. 1:**
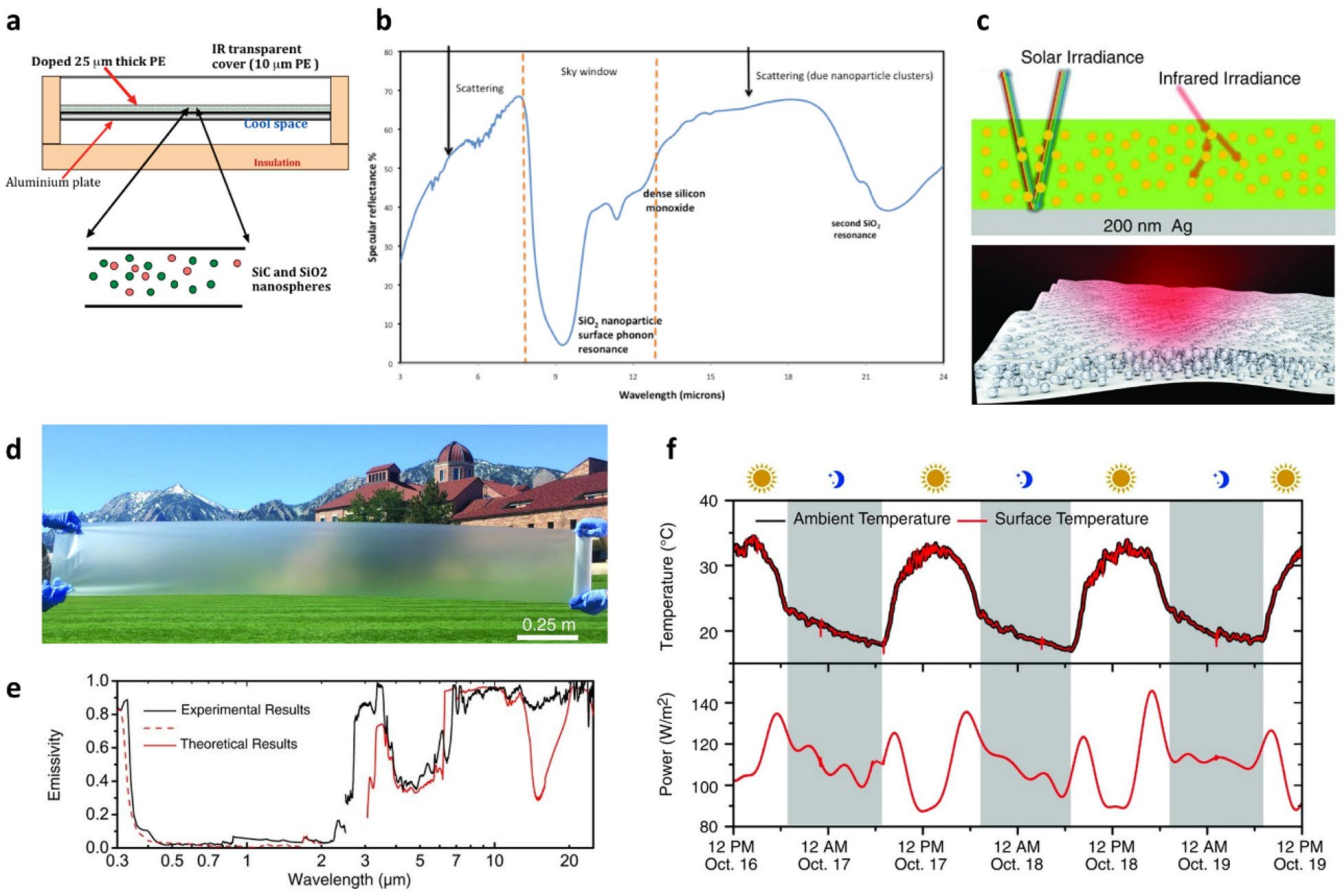
PDRC films consisting of selective thermal emitters and a metal reflective layer. **a** Schematic of an RCM comprising SiO_2_- and SiC-doped PE films. **b** Measured reflectance spectra of RCMs at thermal radiation wavelengths. Reproduced with permission from [64]. Copyright 2010, American Chemical Society. **c** Schematic of SiO_2_–TPX hybrid metamaterial. **d** Photograph of 300-mm-wide hybrid metamaterial film. **e** Emissivity/absorptivity and **f** outdoor temperature measurement results of the hybrid metamaterial. Reproduced with permission from [44]. Copyright 2017, Science

IR emissive materials that possess radiative properties over a wide spectral range in IR exhibit reduced RC performance compared to that of selective emitters in the atmospheric transparent window owing to the reabsorption of radiated heat at wavelengths outside the 8–13 μm wavelength range [24]. Therefore, NP- and MP-based RCMs that can exhibits selective emissivity in narrow spectral regions within the atmospheric window are desirable for achieving efficient PDRC. For example, when inorganic NPs of suitable size are utilized as RCMs, it is possible to ensure high emissivity in the atmospheric window owing to the collective excitation of surface phonon resonance NPs [64]. Moreover, owing to the Mie-scattering of NPs, incident light resonates with NPs and is scattered strongly, which leads to a high reflectance in the solar spectrum [63]. Inorganic particle-based RCMs can be prepared as pristine particle films or as polymeric composites blended with NPs. In general, a high solar reflectance can be achieved by depositing metallic reflective layers such as silver and aluminum. For example, Gentle et. al. developed RCMs by using silicon dioxide (SiO_2_) and silicon carbide (SiC) NPs embedded in polyethylene (PE) film as selective thermal emitters and a aluminum layer as a solar reflector (Fig. 1a) [64]. In these RCMs, phonon-resonant SiO_2_ and SiC NPs were collectively excited by phonon-polariton resonance resulting in enhanced IR emission and increased optical path length in the atmospheric transparent window (Fig. 1b). According to their experimental results, the RCMs exhibited an average emissivity of 95% in the atmospheric transparency window and achieved sub-ambient cooling of 17 ℃ in the nighttime. This approach facilitated simple and inexpensive fabrication of RCMs over a large area and on various substrates. Various types of inorganic particles (TiO_2_, SiO_2_, SiC and Al_2_O_3_) have been utilized to prepare selective IR emitters. Zhai et al. demonstrated PDRC by using randomized glass-polymer hybrid films [44]. Their PDRC films consisted of a 50 μm thick-polymethylpentene (TPX) films and 8-μm-diameter SiO_2_ MPs that were randomly distributed in the films (Fig. 1c). Ag layer was deposited on the film as a solar reflector. Cost-effective, scalable fabrication of more than 300 mm-wide flexible PDRC films polymer was demonstrated (Fig. 1d). These films exhibited a high emissivity of 93% in the atmospheric transparency window owing to surface phonon resonance of SiO_2_ MPs and the high solar reflectivity of 96% of the silver coating (Fig. 1e). In outdoor measurements, this PDRC films yielded a cooling power density of 93 W/m^2^ between 11:00 and 14:00 and an average cooling power density of 110 W/m^2^ over 3 days (Fig. 1f). Liu et al. introduced Al_2_O_3_ NPs with polymer films as IR emitters [65]. They used Al_2_O_3_ NPs mixed with transparent dipentaerythritol penta-hexa-acrylate (DPHA) films as IR emitters and metallic Ag layers as solar reflectors The molecular vibration of DPHA and the phonon mode of Al_2_O_3_ NPs provide high emissivity in the atmospheric transparency window, resulting in a high IR emissivity of 91.63% and solar reflectivity of 94.65% owing to the aforementioned solar reflector. This IR emitter led to a sub-ambient temperature drop of 10.35 ℃ in the daytime.

**Fig. 2:**
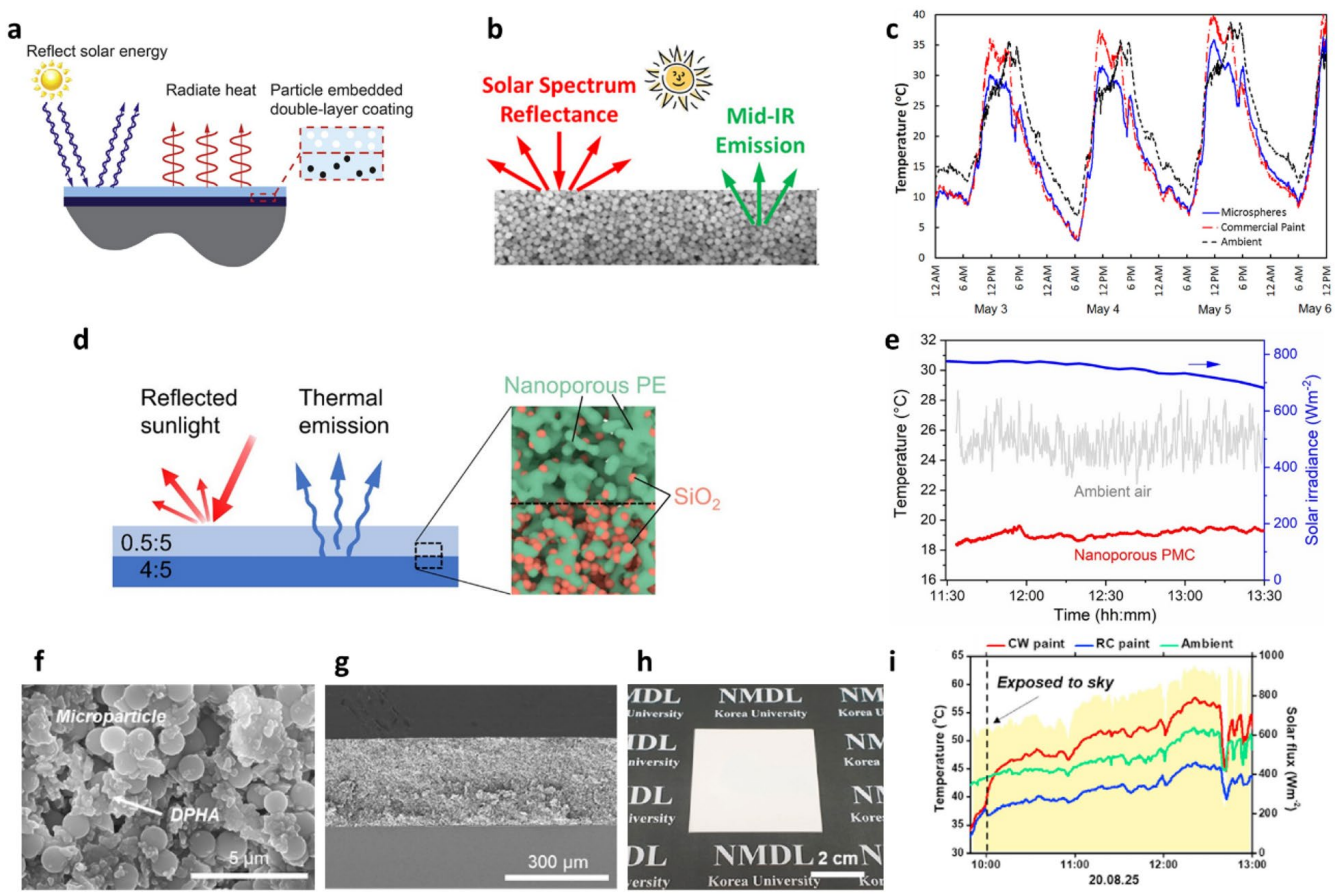
**a** PDRC films without a metal reflective layer. Schematic of a PDRC film composed of a particle-embedded double-layer coating. Reproduced with permission from [69]. Copyright 2017, Elsevier. **b** Conceptual description of particle-based PDRC films. **c** Outdoor temperature profiles of SiO_2_ MP films and a commercial paint relative to ambient temperature. Reproduced with permission from [56]. Copyright 2018, American Chemical Society. **d** Schematic of nanoporous PE film doped with SiO_2_ NPs for PDRC, and **e** its temperature profile under solar irradiation. Reproduced with permission from [62]. Copyright 2021, American Chemical Society. **f** Top and **g** cross-sectional SEM images, and **h** photograph of RC paint on glass substrate. **i** Temperature profiles of commercial CW paint, RC paint, and ambient temperature in the daytime. Reproduced with permission from [52]. Copyright 2021, Elsevier

Although the metal reflective layer of RCMs provides high solar reflectance, it leads to several problems in practical application, such as its inherent UV absorption properties, low chemical stability under oxidation, and high cost of deposition. Therefore, extensive studies have been conducted to achieve high solar reflectance and high IR emissivity in the absence of a metal reflective layer. The energy bandgap and refractive index of a material influence its fundamental optical properties, and they are directly related to the chemical composition, size, structure, and crystal phase of the material [66, 67]. To achieve high solar reflectivity, materials should be designed to have a wide bandgap and high refractive index to ensure that solar irradiation is not absorbed. Owing to their high size tunability and high refractive index, inorganic NPs provide high solar reflectivity without requiring metal reflectors, which allows for simple, inexpensive, and scalable fabrication of RCMs. Bao et al. demonstrated double-layer RCMs comprising TiO_2_ NP films as the top solar reflector and SiC and SiO_2_ mixtures as the bottom IR-emitting layer [68]. The TiO_2_ NPs have a high refractive index, wide bandgap, and high transparency to IR radiation. The SiC and SiO_2_ NPs have high emissivity across “sky windows” in the optical phonon modes. TiO_2_ NPs with an average radius of 500 nm and SiO_2_ and SiC NPs with average diameters of 50 nm were obtained commercially. All films were deposited by means of spray coating on the substrates by mixing the NPs with isopropyl alcohol. The double-layer RC films exhibited a solar reflectivity of 90.7% without using a metal substrate and an emissivity of 90.11% in the atmospheric window. These films cooled an Al foil by 8 °C in the daytime and by 5 °C in the nighttime. Moreover, they cooled a black surface by more than 30 °C in the daytime without exhibiting the degradation issues associated with metal reflectors. Huang et al. also fabricated double-layer coating comprising TiO_2_ NPs embedded in a polymer matrix (Fig. 2a) [69]. TiO_2_ NPs were incorporated in acrylic resin formed the top solar reflector layer, and carbon black NPs were used in the bottom layer to emit heat into the atmospheric transparency window. The dependence of spectral reflectivity, emissivity, and RC performance were investigated. When TiO_2_ NPs with a radius of 200 nm were used, more than 90% of the incident solar irradiation was reflected, and the average emissivity in the atmospheric transparency window exceeded 90% along most directions. The net cooling power density of these designed RCMs was higher than 100 W/m^2^ during the daytime and higher than 180 W/m^2^ during the nighttime at ambient temperature. Atiganyanun et al. developed a paint-format MPs coating for PDRC (Fig. 2b) [56]. Randomly packed SiO_2_ MPs with a diameter of 2 μm were spray-coated on a black substrate to form a white RC coating. The MP coating consisted of SiO_2_ MPs with a relatively low refractive index of 1.46, which may suffer from inefficient light scattering. However, the MP coating exhibited a high reflectivity of 97% owing to the suitable size and fill fraction of SiO_2_ MPs for solar reflection. Furthermore, the MP coating exhibited a high emissivity of 94%, because the porous media provided improved optical impedance matching with air. The MP coating reduced the substrate temperature by 12 °C during the daytime under direct sunlight (Fig. 2c). Composite RC materials composed of a nanoporous PE polymer matrix and SiO_2_ MPs were proposed for daytime RC (Fig. 2d) [62]. By adjusting the nanopore size such that it was comparable to the ultraviolet, visible, and near-IR (NIR) wavelengths, high solar reflectance was achieved without a silver reflective layer. Additionally, the SiO_2_ MPs acted as wavelength-selective emitters, allowing for strong IR thermal radiation in the 8–13 μm wavelength range. With a high solar reflectance of 96% and IR emissivity of 90%, these nanoporous RC films generated a sub-ambient temperature drop of 6.1 °C and cooling power density of 85 W/m^2^ under direct sunlight (Fig. 2e). Chae et al. demonstrated a paint-format MP-polymer composite [52]. RC paint was prepared using an Al_2_O_3_ and SiO_2_ MP mixture, and dipentaerythritol penta-hexa-acrylate (DPHA) was used as the binder (Fig. 2f–h). By mixing the Al_2_O_3_ and SiO_2_ MPs, optical properties favorable for PDRC were realized owing to the high refractive index of Al_2_O_3_ and strong IR emission properties of Al_2_O_3_ and SiO_2_ MPs in the 8–13 μm wavelength range. The size of the MPs was selected in the range of 0.8–1.6 μm, which is comparable to the size required for Mie-scattering in the NIR region within the solar spectrum. The proposed RC paint exhibited a high solar reflectance of 94.1% and emissivity of 93.5% in the atmospheric window, resulting in a sub-ambient temperature drop of 7.9 °C and cooling power density of approximately 100 W/m^2^ (Fig. 2i). Moreover, in an outdoor measurement, the proposed RC paint exhibited a strong PDRC effect compared to that of a commercially available white paint. The proposed paint-format radiant cooler, characterized by the material abundance of Al_2_O_3_ and SiO_2_ and a simple manufacturing process, has excellent potential for PDRC relative to that of commercial paints.

**Fig. 3:**
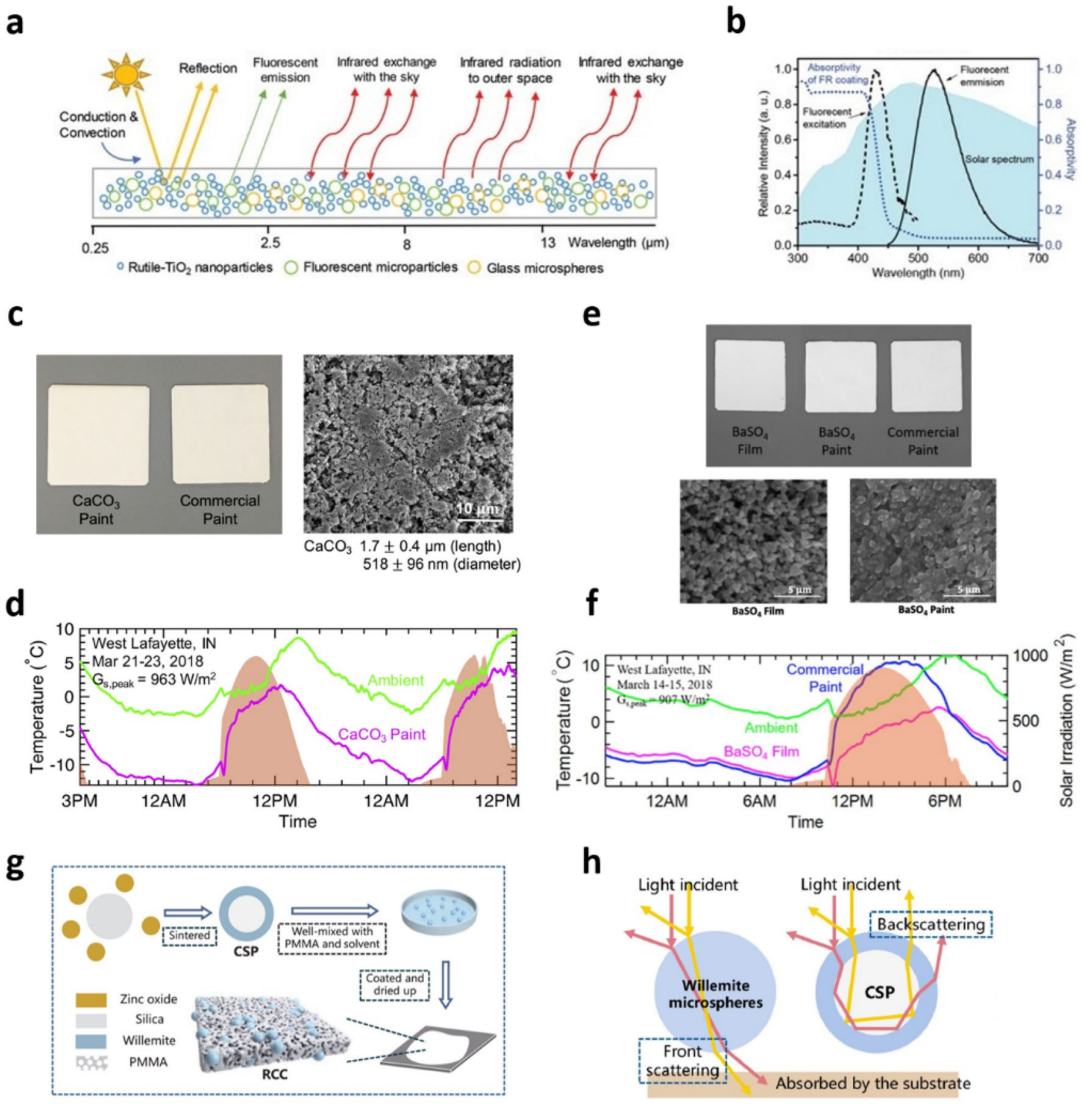
**a** Schematics of PDRC film composed of NPs and MPs acting as solar reflectors, fluorescent particles, and IR emitters in polymer binders. **b** Absorption, fluorescent excitation, and emission spectra of fluorescent MPs to minimize UV absorption of PDRC films. Reproduced with permission from [57]. Copyright 2020, Wiley. **c** Photograph and SEM image of CaCO_3_-acrylic paint along with commercial white paint and **d** outdoor temperature measurement results of CaCO_3_-acrylic paint. Reproduced with permission from [54]. Copyright 2020, Cell Press. **e** Photograph and SEM image of BaSO_4_-based PDRC films and **f** outdoor RC test results of BaSO_4_-acrylic paint compared with the ambient temperature. Reproduced with permission from [51]. Copyright 2021, American Chemical Society. **g** Fabrication process of ZnO-SiO_2_ core-shell particles, and **h** schematics of the light scattering from core-shell structure. Reproduced with permission from [58]. Copyright 2021, Elsevier

Wide-bandgap semiconductors such as TiO_2_ and ZnO offer considerable advantages as solar reflectors in RCMs because these NPs and MPs are commercially available, inexpensive, and exhibit high reflectance in the visible and NIR regions owing to their high refractive indices [23, 70, 71]. However, their absorption properties in the UV region reduce the RC efficiency because there is a non-negligible portion of UV in the solar spectrum, and the absorption of UV energy increases the temperature of the RCMs. This limitation can be overcome by utilizing luminescent spectral converters that absorb solar energy in the UV region and reemit it in the visible or NIR region with high efficiency, thereby preventing an increase in the temperature of the RCMs (Fig. 3a) [57]. Fluorescent pigment RCMs were fabricated by mixing commercially available TiO_2_ NPs, fluorescent MPs, and glass MPs in a polystyrene-acrylic emulsion. Polystyrene-acrylic, TiO_2_ NPs, fluorescent MPs, and glass MPs acted as the polymer matrix, solar reflector, spectral converter, and IR thermal emitter, respectively. The addition of fluorescent MPs significantly reduced the UV absorbance of the TiO_2_ NPs by converting the absorbed sunlight below 450 nm into emission in the visible region (Fig. 3b). This RCM reduced the temperature by 6 °C relative to ambient temperature under sunlight, yielding a cooling power density of 84.2 W/m^2^. Insulating CaCO_3_ and BaSO_4_ NPs or MPs have been used to eliminate the limitation that ZnO and TiO_2_-based paints absorb UV [51, 54]. Although CaCO_3_ has a low refractive index, its broad size distribution and assemblies consisting of large numbers of particles enhance its solar reflection properties [72, 73]. The solar reflectance of RCMs was increased by increasing the CaCO_3_ concentration to induce the optical crowding effect. In addition, the polydispersity of particle size distribution efficiently increases the scattering efficiency over the wide wavelength range of the solar spectrum, which improves the solar reflectance of the films in the solar spectrum (Fig. 3c) [54]. The CaCO_3_-acrylic paint exhibited a high solar reflectance of 95.5%, an emissivity of 94% in the atmospheric window, temperature drop of 1.7 °C relative to ambient temperature, and cooling power density of 37 W/m^2^ (Fig. 3d). A BaSO_4_-acrylic paint for solar reflectors with low solar absorptance owing to its wide optical bandgap (~ 6 eV) was demonstrated (Fig. 3e) [51]. With an appropriate particle size and a broad particle size distribution, BaSO_4_ NP films achieved a solar reflectance of 97.6%, atmospheric window emissivity of 96%, and a cooling of 4.5 °C below ambient temperature with an average cooling power density of 117 W/m^2^ (Fig. 3f). Multicomponent, core-shell type inorganic particles were introduced to improve the solar reflectance and IR emissivity of RCMs [58]. Core-shell particles were synthesized by sintering of a mixture of ZnO NPs and SiO_2_ MPs (Fig. 3g). At a sufficiently high temperature, ZnO diffused into the silica matrix and was converted into the willemite (Zn_2_SiO_4_) phase on the surface of SiO_2_ MPs to form a core-shell type structure. RC films were fabricated by coating the paint slurry composed of these core-shell particles and PMMA binders. The difference between the reflective indices of the core and shell components led to backscattering of the incident solar irradiation, which resulted in higher solar reflectance compared to that of the films composed of a either ZnO or SiO_2_ alone (Fig. 3h). Moreover, the IR optical phonon vibration of Zn-O-Si, Si-O-Si bonds, SiO_4_, and ZnO_4_ groups covered a wide wavelength range of the atmospheric transparency window, which increased the radiative thermal emissivity of the films. These films achieved a solar reflectance of 96%, IR emissivity of 94%, and temperature drop of 4.1 ℃ and 5.3 ℃ in the daytime and nighttime, respectively. Hollow SiO_2_ NPs for RC materials have been reported for high-solar-reflectance and high-thermal-emissivity RCMs [74]. Hollow SiO_2_ NPs were synthesized by means of emulsion polymerization with n-octadecane to form paraffin-SiO_2_ core-shell NPs, followed by calcination at high temperatures to remove the core paraffin. Hollow SiO_2_ NPs were then blended with poly (vinylidene fluoride-co-hexafluoropropylene) (PVDF-HFP) and deposited on the substrate through spray coating to form a white PDRC film. The average solar reflectance of the hollow SiO_2_ NP films was 97.2% in the wavelength range of 0.3–2.5 μm, and their emissivity in the atmospheric window was 94.3% owing to the phonon mode of the Si-O bonds in the hollow SiO_2_ NPs and molecular vibrational modes of the PVDF-HFP polymer binder. Under direct sunlight, this white PDRC film achieved a sub-ambient temperature drop of 6.12 ℃ with a cooling power density of 40.75 W/m^2^.

**Fig. 4:**
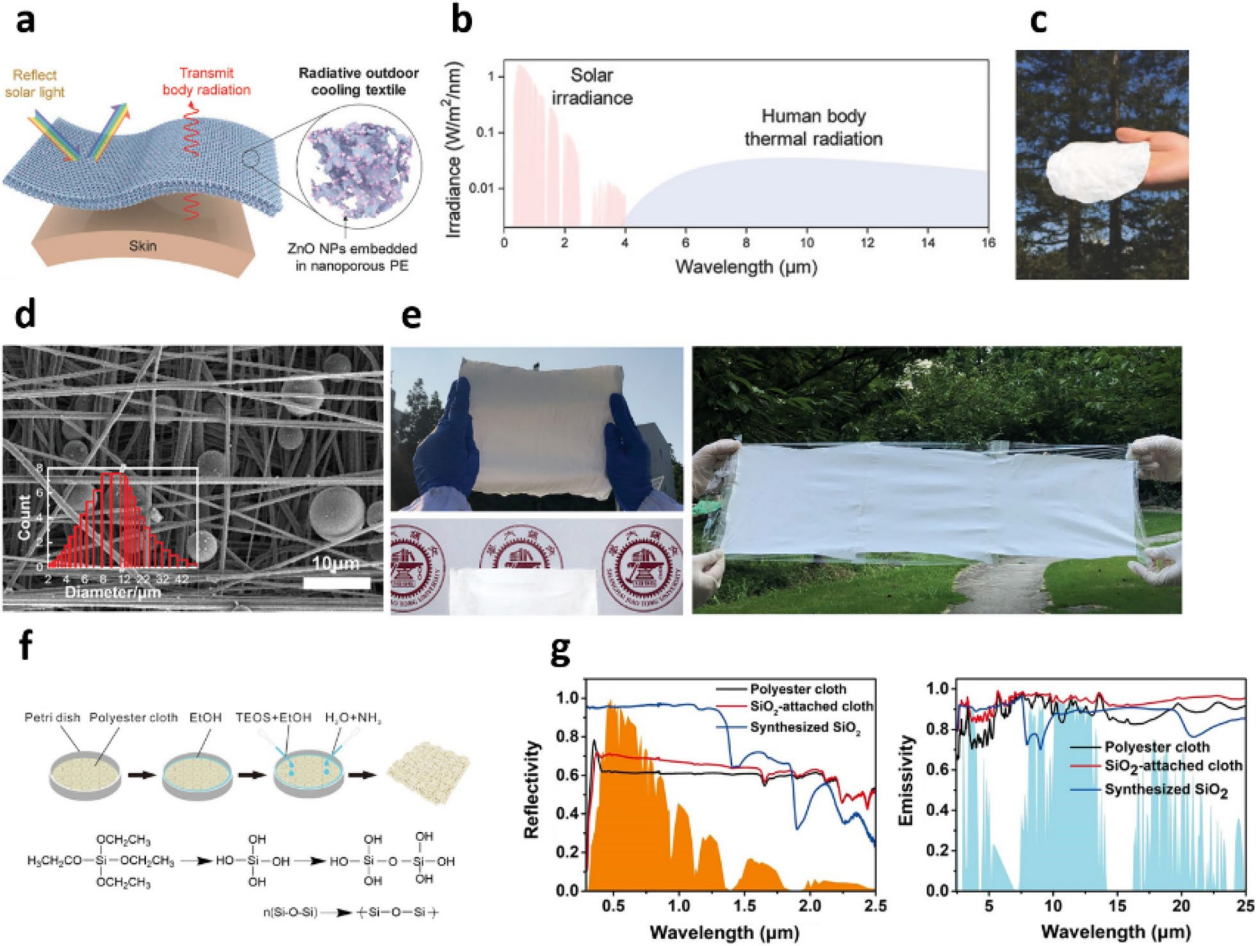
**a** Schematic of ZnO NP-embedded nanoporous PE textile. **b** AM1.5 solar irradiation spectra and thermal radiation of human body simulated using Planck’s law at the skin temperature of 34 ℃. **c** Photograph of ZnO-PE fabric. Reproduced with permission from [50]. Copyright 2018, Wiley. **d** SEM image of hybrid membrane radiator composed of PVDF/TEOS fibers and SiO_2_ MPs. **e** Photograph of scalable flexible hybrid membrane radiator. Reproduced with permission from [59]. Copyright 2020, Wiley. **f** Fabrication process of SiO_2_-attached fabrics. **g** Spectral reflectivity and emissivity of SiO_2_-attached fabrics and raw materials. Reproduced with permission from [60]. Copyright 2021, American Chemical Society

Furthermore, the application of RCMs to fabrics or textiles has been reported by researchers by incorporating NPs or MPs in the matrix to allow for efficient personal thermal management [21]. These RC fabrics have tremendous potential in many emerging applications such as packaging [61, 75], transportation [76, 77], vehicle cooling [16, 22], and smart devices [48, 50, 59, 60]. Owing to the high dispersity of colloidal NPs and MPs in various types of solvents or polymer mediums, RC particles can be easily incorporated into fabric or textiles. Cai et al. demonstrated a smart fabric with spectrally selective radiation properties for passive outdoor personal cooling [50]. Their smart fabric consisted of ZnO NPs embedded in nanoporous PE, as displayed in Fig. 4a. In this design, human skin acted as the IR thermal emitter with an IR emissivity of 98%. At a skin temperature of 34 ℃, the human body emits thermal radiation in the range of 7–14 μm, which overlaps with the IR transparency window of the Earth’s atmosphere (Fig. 4b). Therefore, the heat radiated from the human body can be dissipated to the cold outer space through the atmospheric window. PE composed of aliphatic C–C and C–H bonds was used as an IR transparent polymer matrix to transmit thermal radiation out from the human body. By contrast, ZnO NPs, which have a high refractive index and little absorption in the visible (400 nm) to mid-IR wavelengths (16 μm), were utilized as the solar-reflecting material. The fabric was prepared by mixing commercial ZnO NPs (particle sizes of 300–800 nm) with PE in paraffin oil at 200 ℃. The mixture was then melt-pressed into a thin film at 70–100 ℃, and the paraffin oil was subsequently extracted from the film by using methylene chloride. The prepared fabric with a thickness of 150 μm was white in color under sunlight (Fig. 4c). This smart fabric had a high reflectivity exceeding 90% in the solar spectrum and a high transmissivity of 80% in the 7–14 μm wavelength range, in which thermal radiation from the human body is centralized. In real-time outdoor measurements, the smart fabric prevented overheating by 5–13 ℃ compared to normal fabric under direct sunlight. Wang et al. fabricated a flexible hybrid membrane thermal radiators by means of a facile electrospinning process [59]. SiO_2_ MPs measuring 6–14 μm in diameter were randomly distributed in polyvinylidene fluoride/tetraethoxysilane (PVDF/TEOS) fibers with numerous nanopores (Figs. 4d and e). PVDF/TEOS fibers have strong mid-IR absorption owing to the molecular vibration modes of the C-F and Si-O bonds in PVDF and TEOS, respectively. The SiO_2_ MPs further enhance this mid-IR emissivity because SiO_2_ has strong mid-IR absorption due to the phonon-polariton resonance of the Si-O bond at 9.7 μm. Furthermore, nanopores with diameters of 100–200 nm in the fibers increased the high solar reflectance owing to Mie-scattering. Consequently, this membrane radiator exhibited an average IR emissivity of 96% and reflected 97% of solar irradiance in the absence of a reflective metallic layer. Moreover, it caused a temperature drop of 6 ℃ under direct sunlight with an average radiative cooling power density of 61 W/m^2^. Fabric-format RCMs can be fabricated simply through in situ synthesis of SiO_2_ NPs in polyester cloth [60]. By using the Stober method, which is well known as a synthetic method for silica, SiO_2_ NPs can be formed in-situ on polyester cloth through hydrolysis and condensation of the silane monomer in the presence of ammonia solution (Fig. 4f). Strong phonon-polariton resonance in the surface and intrinsic vibration modes of SiO_2_ MPs lead to high emissivity in the atmospheric window. Furthermore, SiO_2_ NPs measuring 200–450 nm in diameter effectively reflected radiation in the vis-NIR region owing to their Mie-scattering ability. The SiO_2_-attatched fabrics have a high emissivity of more than 95% in the atmospheric window and a relatively high reflectivity of ~ 70% in the visible-NIR region (Fig. 4g). Under direct sunlight, these fabrics achieved a temperature drop of 11.2 ℃ with a cooling power density of 45 W/m^2^. Zhang et al. demonstrated the fabrication of a RC fabric composed of PVDF-HFP nanofibers and SiO_2_ NPs by means of electrospinning [61]. The IR absorption peaks of the PVDF-HFP fabric were located inside the atmospheric window, which made it a suitable candidate for RCMs. Moreover, PVDF-HFP has excellent mechanical properties and flexibility, which help it serve as an excellent RC fabric. This RC fabric has a high solar reflectance of 97% and a high emissivity of 94% in the atmospheric window, and it achieved excellent RC effects under direct sunlight.

## Colored RCMs

To utilize PDRC in residential and commercial applications for aesthetic purposes, the coloration of RCMs should be considered. However, most RC materials have white or metallic colors for achieving high solar reflectance because colors of RCMs originate from the absorption of visible solar light, which increases their temperature. Therefore, to implement colors into RCMs, new strategies to minimize the absorption of solar energy while maintaining apparent colors are needed. Colloidal NPs and MPs are ideal platforms for engineering the optical properties of materials by changing their size, shape, composition, and crystal structure to realize novel optical properties that distinct from those of their bulk counterparts. Many strategies for designing colored RCMs by using colloidal particles have been proposed and experimentally demonstrated, for example, utilizing the structural colors of colloidal assemblies [78] or their plasmonic properties [79, 80], minimizing solar absorption by selectively narrowing absorption in the visible region [81, 82], and using luminescent wavelength convertors.

**Fig. 5:**
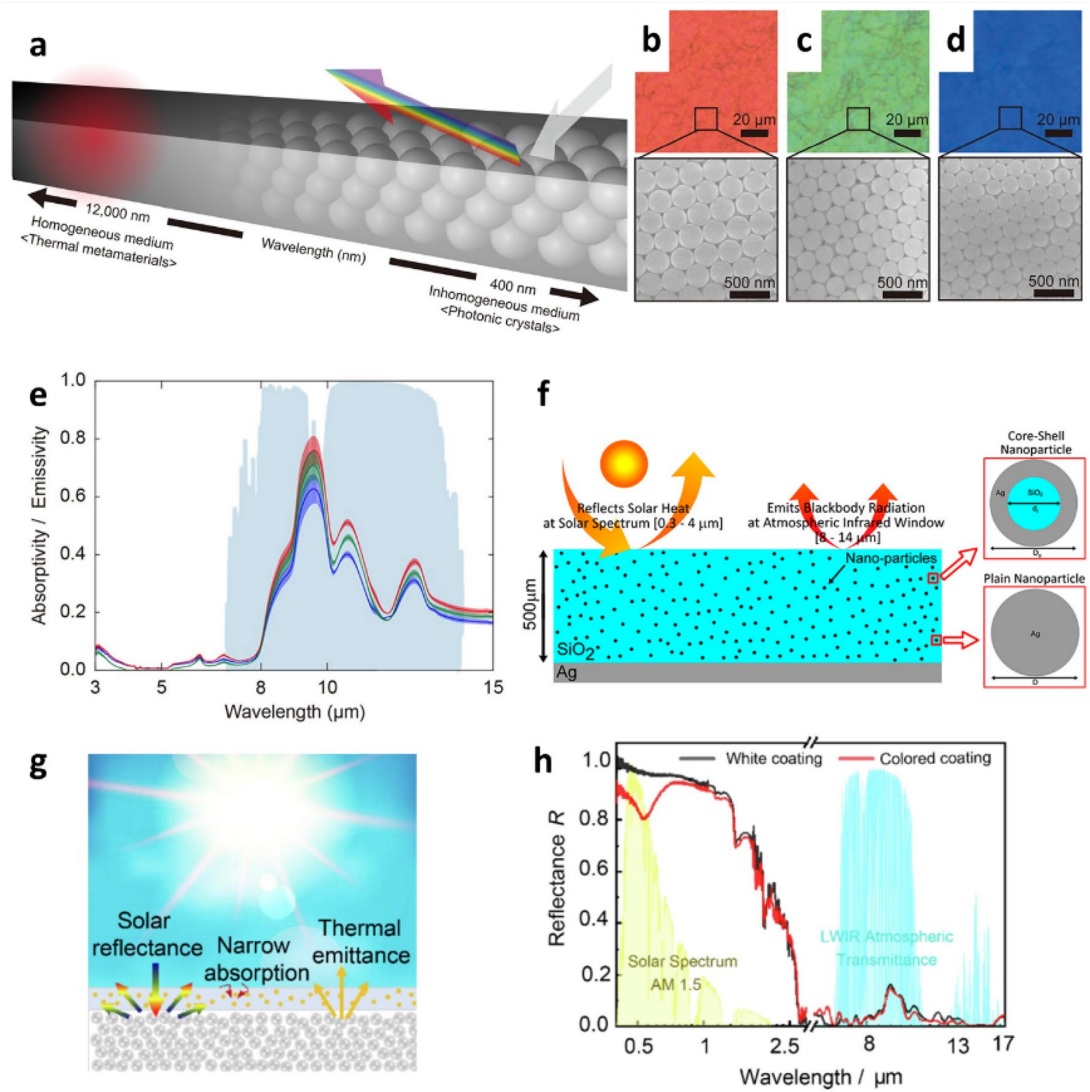
**a** Design principle of colored RCMs using photonic opal crystals. Bright-field optical microscopy (top) and SEM (bottom) images of **b** reddish, **c** greenish, and **d** bluish opals. **e** Experimentally measured absorptivity/emissivity of colored opals in the mid-IR regions. Reproduced with permission from [78]. Copyright 2020, American Chemical Society. **f** Colored coating composed of SiO_2_-Ag core-shell NPs embedded in silica. Reproduced with permission from [79]. Copyright 2020, American Chemical Society. **g** Schematic of bilayer colored PDRC coating consisting of a layer composed of plasmonic spheres and another layer composed of randomly dispersed dielectric spheres. **h** Spectral reflectance of white SiO_2_ and colored coatings. Reproduced with permission from [80]. Copyright 2022, Elsevier

The structural colors of colloidal particles originate from the interaction between incident light and the periodic structures of particles such as reflection, scattering, interference, and diffraction [83, 84]. When colloidal particles are arranged as photonic structures, RCMs exhibit structural colors owing to the controlled scattering of solar irradiation depending on its wavelength. For example, RCMs with structural colors due to Bragg diffraction have been demonstrated in the literature (Fig. 5a) [78]. Opals are close-packed face-centered cubic crystals that are self-assembled using colloidal particles. The primitive cells of opals exhibits iridescent colors owing to Bragg diffraction, where the size of the colloidal particles is comparable to the wavelength of visible light [85, 86]. For the synthesis of colored RCMs, SiO_2_ NPs are synthesized using the Stober method. These SiO_2_ NPs are then self-assembled on a silicon wafer coated with a polydimethylsiloxane (PDMS) adhesion layer. By varying the size of the SiO_2_ NPs, the position of the bandgap in a photonic structure can be precisely tailored over the entire visible spectrum, resulting in vivid structural colors (red, green, and blue) (Figs. 5b–d). Furthermore, opals absorb and emit thermal energy in the mid-IR region owing to the Si-O vibrations in SiO_2_ NPs (Fig. 5e), which allows opals to act as effective IR thermal radiators in the atmospheric window. Red-, green-, and blue- colored RCMs reduce the temperature of the Si substrate by 13 ℃, 11 ℃, and 9 ℃, respectively, under sunlight. The utilization of surface plasmon resonance effects for the colorization of RCMs has been reported as well. Surface plasmon resonance refers to the collective oscillation of free electrons on the surface of a material owing to resonance between free electrons and incident electromagnetic waves [87, 88]. The resonance frequencies are influenced by the size, shape, and compositions of the NPs, as well as the dielectric constant around the NPs. Especially, metallic NPs such as those of gold, silver, and copper have many free electrons, and for this reason, their resonance frequencies are in the visible wavelength region. Surface plasmon resonance can induce light scattering at the resonance frequency, which results in characteristic reflection colors. Colored RCMs that use plasmonic effects were fabricated using silica-silver core-shell NPs embedded in an SiO_2_ or a PDMS matrix deposited over a silver reflector (Fig. 5f) [79]. These composite coating exhibited a color owing to their narrow extinction properties within the visible region because of the localized surface plasmon resonance of silver NPs. The spectral position of the surface plasmon resonance was tailored by changing the radii of the core and shell, which changed the apparent color. These core-shell NPs exhibited selective absorption peaks in the entire visible range when the shell thickness was changed while maintaining a constant core size. Although outdoor RC experiments were not conducted, the results of a numerical study on colored RCMs consisting of plasmonic NPs highlighted the possibility that these colored RCMs could have a positive radiative cooling power density when utilizing NPs of an appropriate size, concentration, and core and shell radii. For example, the red-colored RCM theoretically exhibited a radiative cooling power density of 28–117 W/m^2^ depending on the concentration of plasmonic Ag NPs in the films. However, owing to the non-negligible absorption of Ag NPs, the RC power density decreased as the concentration of Ag NPs in the films increased. M. Chen et al. experimentally demonstrated colored RCMs by using the surface plasmonic effect (Fig. 5g) [80]. In the underlayer of a colored RC coating, SiO_2_ MPs simultaneously served as a solar reflector and thermal radiator. Plasmonic NPs selectively absorbed the photons with narrow resonance wavelengths for coloration, while no absorption occurred at other wavelengths to minimize solar heating. This colored RC coating exhibited a solar reflectivity exceeding 82%, high atmospheric emissivity exceeding 93%, and temperature drops of 7.5 ℃ and 5.1 ℃ for the films covered with Au and Ag plasmonic NPs, respectively.

**Fig. 6:**
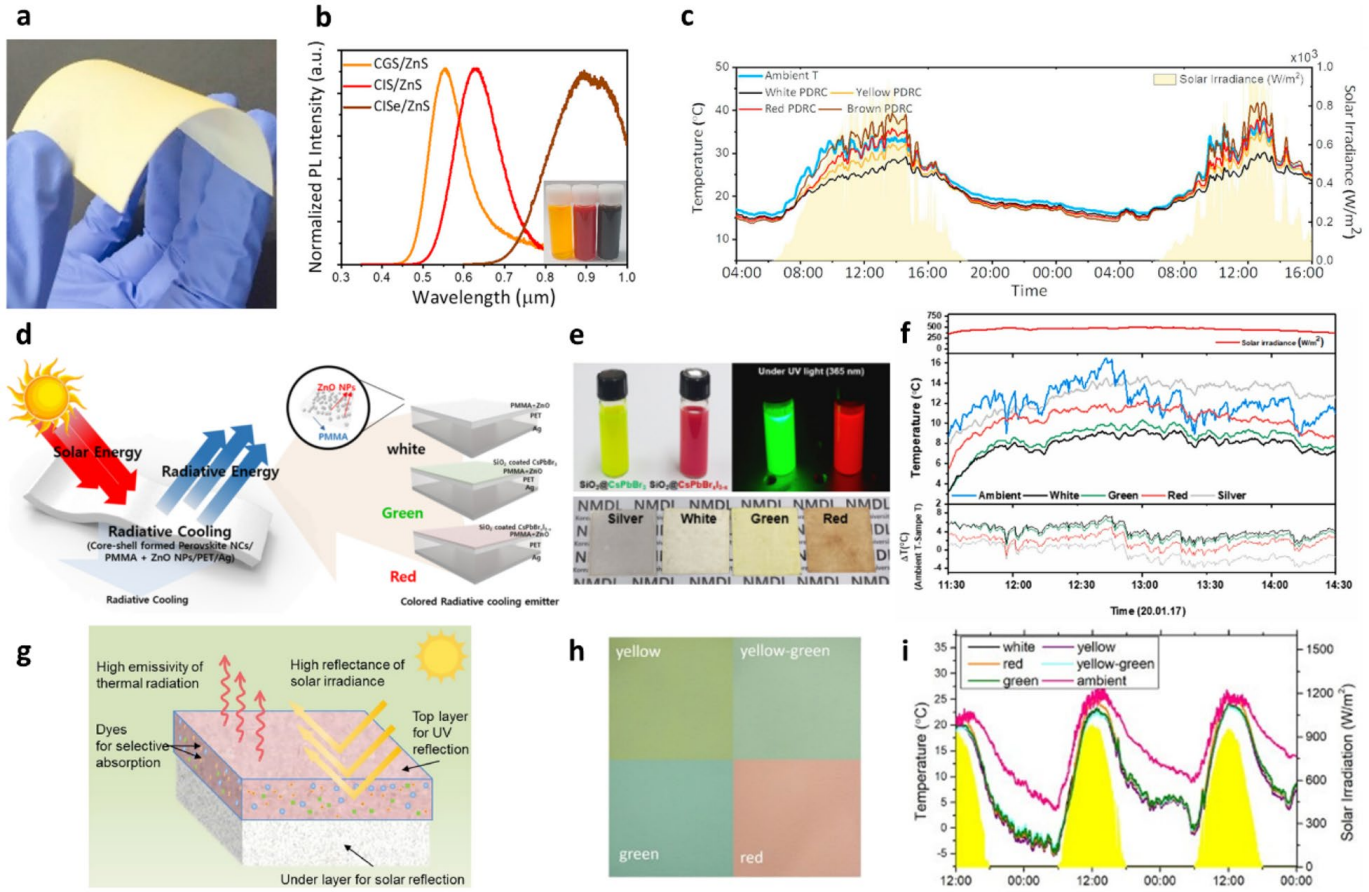
**a** Photograph of yellow PDRC film coating on a flexible substrate. **b** Normalized PL spectra and photographs of CuGaS_2_@ZnS, CuInS_2_@ZnS, and CuInSe_2_@ZnS core-shell QD solutions. **c** Temperature profiles of white, yellow, red, and brown PDRC films compared to the ambient temperature. Reproduced with permission from [74]. Copyright 2021, Elsevier. **d** Schematic of colored RCMs with silica-embedded perovskite NCs/PMMA + ZnO/PET/Ag films. **e** Photos of perovskite NCs in hexane and colored RCM films. **f** Daytime temperature profiles of colored RCMs. Reproduced with permission from [81]. Copyright 2021, Elsevier. **g** Schematic illustration and **h** photographs of colored RC coatings with a bilayer structure. **i** Outdoor temperature profiles of colored RC coatings compared to the ambient temperature. Reproduced with permission from [92]. Copyright 2022, Elsevier

Colored RCMs based on luminescent spectral converters have been reported [74, 81, 82]. Luminescent materials absorb high-energy photons and re-emit them in low-energy region with a high conversion efficiency. In the case of colored RCMs, solar energy absorption can be minimized when the photons absorbed in the visible wavelength region are effectively converted to low-energy photons and emitted by spectral converters. Luminescent quantum dots (QDs) serve as ideal platforms for spectral converters in colored RCMs owing to their high extinction coefficient, high conversion efficiency, color purity, and photostability [89–91]. In addition, the apparent colors of QDs can be tailored readily by changing their size, shape, and composition, which leads to versatile design capabilities for the development of colored RCMs. Yoon et al. reported colored RCMs fabricated by means of sequential solution-based deposition of colloidal QDs and hollow SiO_2_ NPs (Fig. 6a) [74]. To achieve high solar reflectance and IR emissivity, hollow SiO_2_ NPs mixed with polymer binders were deposited on the substrates. Subsequently, heavy-metal-free Cu-based I-III-IV ternary QDs were deposited on the top of the hollow SiO_2_ NP films to form colored RCMs. The colors of these RCMs were tailored by controlling the composition of the QDs. The CuGaS_2_, CuInS_2_, and CuInSe_2_ QDs exhibit yellow, red, and brown colors, respectively, owing to the partial absorption of visible wavelengths higher than the bandgap of the QDs (Fig. 6b). To improve conversion efficiency, all the QDs were overcoated with a thin layer of ZnS to form a core-shell structure. The emissivity of these colored RC films exceeded 94% in the 8–13 μm wavelength range, similar to that of the white RC film composed of hollow SiO_2_ only. While the white RC film exhibited a solar absorptivity of 2.3% in the 0.3–2.5 μm wavelength range, the colored RC films exhibited higher solar absorptivities because of the presence of QDs (yellow: 6.9%, red: 8.1%, and brown: 24.6%). The colored RCMs continued to exhibit PDRC properties despite strong absorption in the visible wavelength region. In outdoor temperature measurements, the temperature of the colored RC films was lower than the ambient temperature under sun light (white: 6.12 ℃, yellow: 3.25 ℃, red: 0.51 ℃, and brown: − 3.24 ℃) (Fig. 6c). This result demonstrated that the colored RC films simultaneously achieved efficient RC performance and coloration through absorption and re-emission of visible solar energy to the environment for minimizing solar energy absorption. Colored RCMs were easily fabricated by sequentially spray-coating colloidal NP inks on various substrates, which allowed for facile and inexpensive fabrication of colored RC materials over a large area, even on existing structures such as buildings and vehicles. In addition, all the NPs were composed of non-toxic and Earth-abundant elements, which is essential for preventing environmental and health issues. Colored RCMs that employ silica-embedded perovskite NPs were demonstrated on an Ag reflector [81]. These colored emitters consisted of several layers, including an Ag layer as the solar reflection layer, flexible polyethylene terephthalate (PET) substrate as the broadband mid-IR emitting layer, and ZnO NPs in poly(methyl methacrylate) (PMMA + ZnO) as the solar scattering layer (Fig. 6d). Lead halide perovskite QDs were used for coloration of these RCMs. Two types of perovskites NCs, CsPbBr_3_ and CsPbBr_x_I_3-x_, embedded in an SiO_2_ shell were subsequently deposited to form the green and red colors, respectively (Fig. 6e). Surface passivation with silica shell improved the chemical stability and photon conversion efficiency of these RCMs. Furthermore, the silica shell may be able to improve the cooling property of RCMs owing to its mid-IR emission properties. These colored emitters exhibited high absorptivity at the wavelength of 330 nm, which was assigned to the ZnO NPs (3.37 eV bandgap). The green- and red-colored emitters exhibited light absorption near 520 nm and 650 nm, respectively, to produce the respective colors. Because of the PET substrate used in them, these colored emitters exhibited high broadband emissivity in the mid-IR range. In outdoor measurements, the white, green, and red emitters exhibited sub-ambient cooling of 4.2, 3.6, and 1.7 ℃, respectively, during the daytime (Fig. 6f).

Although colloidal semiconducting QDs are promising platforms for RCM coloration, several issues hinder their practical applications, such as difficulty of mass production, high cost, and poor stability under outdoor conditions. Inorganic luminescent phosphors composed of transition metals or lanthanide elements offer huge advantages in terms of mass production, relatively low cost, and structural stability for colored RCMs. J. Xu et al. reported colored RCMs composed of inorganic luminescent phosphors [92]. Colored RCMs were fabricated with a bilayer structure, in which the bottom layer was a TiO_2_-based polyacrylic resin and the top layer was a BaSO_4_ film (Fig. 6g). The TiO_2_-based acrylic resin strongly reflected visible and NIR wavelengths to minimize solar heating. Meanwhile, the BaSO_4_ top layer with phosphors exhibited highly selective narrowband absorption over visible wavelengths, which caused it to show specific colors. Commercially available Y_3_Al_5_O_12_: Ce, Y_3_(Al,Ga)_5_O_12_: Ce, SrSiO_4_: Eu, and CaAlSiN_3_: Eu inorganic phosphors were used as yellow, yellow-green, green, and red colorants, respectively (Fig. 6h). The solar reflectance of the proposed colored RCMs exceeded 0.90, and the sub-ambient temperature drops of the yellow, yellow-green, green, and red coatings were 2.5, 2.7, 2.2, and 0.6 ℃, respectively, under direct sunlight (Fig. 6i).

## Self-adaptive RCMs

Most of RCMs exhibit a single property and a given cooling power. Recently, many researchers have attempted to incorporate self-adaptive properties and proposed dynamically tunable IR adaptive textiles [93, 94] and temperature-adaptive radiative coating by using phase change materials (PCMs) [95–97]. Because there is considerable variation in outdoor temperature between daytime and nighttime, the regulation of thermal power depending on the environmental temperature allows for selective cooling at high temperature and more efficient thermal management [98]. According to Kirchhoff’s Law, the spectral emissivity of vanadium dioxide (VO_2_) decreases as its spectral reflectance increases [99].

$${\varepsilon }_{\lambda ,\theta }={\alpha }_{\lambda ,\theta }; {\alpha }_{\lambda ,\theta }+ {\rho }_{\lambda ,\theta }=1; {\varepsilon }_{\lambda ,\theta }=1-{\rho }_{\lambda ,\theta }$$

${\varepsilon }_{\lambda ,\theta }$, ${\alpha }_{\lambda ,\theta }$, and ${\rho }_{\lambda ,\theta }$ denote normal spectral emissivity, absorptivity, and reflectivity, respectively. VO_2_, a PCM, has tunable optical and thermal properties that can be attributed to a phase transition called metal–insulator transition (MIT) at a critical temperature T_c_ (68 °C). VO_2_ has a monoclinic structure and a relatively IR-transparent state below its T_c_, but it transforms into a metallic tetragonal structure with a relatively IR-reflective state above its T_c_. In other words, VO_2_ has switchable reflectivity and transmissivity in the IR and visible ranges, and the tunability of its IR emissivity in radiative cooling has been demonstrated [100]. The existing studies have mainly focused on manufacturing VO_2_ by using different dielectric spacer layers and optimizing the device geometry to achieve dynamic cooling performance [101] because VO_2_ has disadvantages such as absorption of visible light and difficulty in large-area application. Latent heat across the MIT is 60 kJ·kg^−1^ for bulk crystal VO_2_ (or 5 ± 0.8 kJ·mol^−1^) [102] and 16 kJ·kg^−1^ for the thin-film VO_2_ [103]. This latent heat associated with the solid–solid transition of VO_2_ is not adequate for heat-storage and thermal management applications. However, because of the drastic change in its optical properties along with MIT, VO_2_ can be utilized as a building block to regulate the optical properties of RCMs [101]. Although VO_2_ is a negative emittance switching material (i.e., emissivity decreases as temperature increases), its emissivity can be modified by means of interference with highly reflective substrates. In addition, the significant change in the emissivity and desirable emissivity of metallic VO_2_ in the atmospheric window region is the reason for its high potential as an RCM. For instance, its IR absorption/emissivity can be enhanced by means of Fabry–Perot interference from mirror-cavity-mirror multilayered structures [104]. The ultra-thin VO_2_ film was used to tune the resonance frequency in the NIR [105], and owing to its excellent phase transition behavior, the RCM can act either as an absorber or emitter depending on the phase of VO_2_ [106, 107].

By utilizing the tunable optical and thermal properties of VO_2_, various types of self-adaptive RCMs were designed for tunable thermal management, although most of them were fabricated by means of chamber-based thermal deposition of VO_2_ layers [108, 109]. Tang et al. developed a temperature-adaptive radiative coating by using the strongly correlated electron material W_x_V_1-x_O_2_, the transition temperature of which was tailored to ~ 22 °C (x = 1.5%) [104]. In the 8–13 µm atmosphere transparency window, the insulator state of W_x_V_1-x_O_2_ was transparent to the IR at T < T_c_, and then, sky-window IR radiation was reflected using an Ag mirror. By contrast, metallic W_x_V_1-x_O_2_ exhibited high absorption in the atmosphere window. The solar absorptance (0.3–2.5 um) was not high at ~ 0.25, although its sky-window emittance (8–13 µm) increased from ~ 0.20 in the insulator state to ~ 0.90 in the metallic state. The actual outdoor performance of the temperature-adaptive radiative coating indicated the strong RC performance of W-doped VO_2_. When the ambient temperature was less than T_c_, the temperature of the temperature-adaptive radiative coating was close to the ambient temperature. In the daytime, the solar absorptance and thermal emittance of the temperature-adaptive radiative coating in the metallic state were 0.25 and close to 0.90, respectively. After daytime, the temperature and thermal emittance were similar to those in the initial state, indicating the reversibility of these properties.

**Fig. 7:**
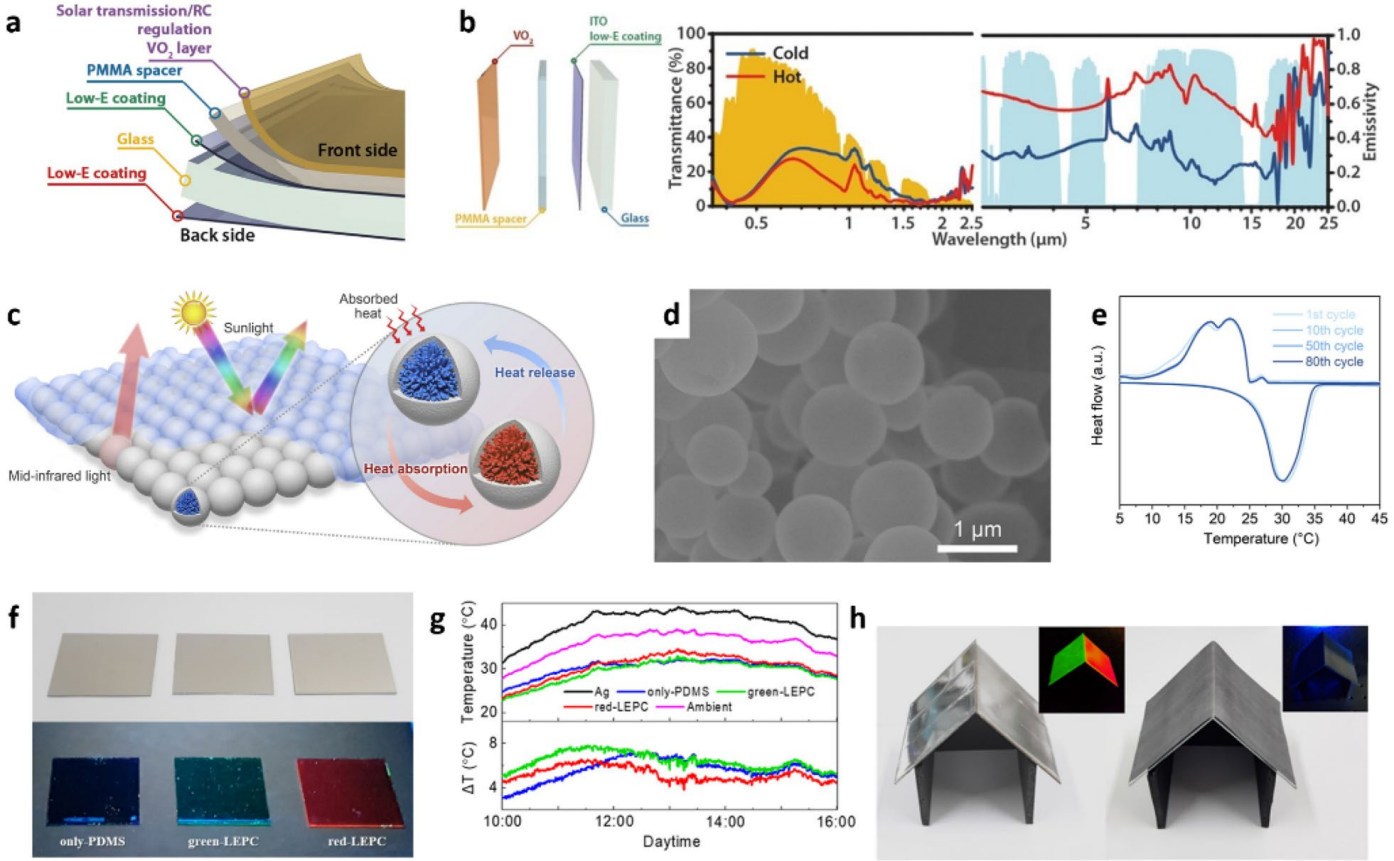
**a** Schematic structure and **b** spectral transmissivity and emissivity of VO_2_ smart window. Reproduced with permission from [110]. Copyright 2021, Science. **c** Schematic diagram, **d** SEM image, and **e** thermal cycling performance of SiO_2_-PCMs MPs. Reproduced with permission from [114]. Copyright 2022, Cell Press. **f** Photos of various RC devices under indoor light (upper) and UV lamp (below). **g** Daytime outdoor temperature and difference between ambient temperature and device temperatures. **h** Artificial houses equipped with a light-emitting cooling roof and conventional roof. Reproduced with permission from [116]. Copyright 2020, American Chemical Society

In addition, the use of colloidal VO_2_ NPs for designing temperature-adaptive RCMs has been reported recently, which allows for facile and large-scale fabrication of PDRC films with comparable modulation performance (Δ ${\upvarepsilon }_{\mathrm{W}}$ > 0.8) [110]. An RC smart window was prepared through a sequential spin-coating process to form VO_2_/PMMA spacer/indium tin oxide (ITO) glass stack (Fig. 7a). The VO_2_ NPs were dispersed in the PMMA solution and spin-coated onto the PMMA spacer to provide the functionality of solar transmission modulation (ΔT_sol_) and RC modulation. The PMMA layer served as spacer owing to its high solar and long-wave IR transparency (LWIR), and the ITO glass was used owing to its high visible transmittance and low LWIR. These structures provided weak absorption in the long-wave IR region at low temperatures but caused strong Fabry–Perot resonance, which led to enhanced LWIR absorption with the metallic VO_2_ layer, at high temperatures. The RC window exhibited high luminous transparency (T_lum_) with a large ΔT_sol_ (9.3%) and an LWIR emissivity difference (Δε_LWIR_) of 0.4, where ΔT_sol_ is the difference in T_sol_ between low and high temperatures, and Δε_LWIR_ is the difference in long-wave IR emissivity between low (ε_LWIR-L_) and high temperatures (ε_LWIR-H_) (Fig. 7b). Owing to the presence of VO_2_ NPs, The RC smart window automatically regulated the RC capability through its tunable IR emissivity depending on changes in the ambient temperature. At both low and high temperatures, the RC smart window exhibited high transparency in the visible range for residential and aesthetic purposes. As the temperature increased, to prevent solar heating, the RC smart window exhibited relatively low NIR transmittance compared to the low temperature, and achieved high emissivity within the atmospheric window. When it was cold outside, the RC smart window exhibited low emissivity in the atmospheric window to suppress RC capability while enhancing NIR transmittance. VO_2_ exhibited significant absorption in visible wavelength region of the solar spectrum, which dramatically reduced the RC performance. If the solar spectrum can be reflected or filtered, solar energy absorption by VO_2_ can be effectively minimized.

Although the PCM VO_2_ is an efficient building block for regulating RC properties, high absorption in the visible light region is a critical drawback of VO_2_ in terms of PDRC applications because this intrinsic absorption property increases the film temperature. To prevent this problem, Ono et al. demonstrated VO_2_–based self-adaptive RCMs by means of selective filtration of solar spectrum in the top layer of RCMs [111]. These self-adaptive RCMs had a bilayer structure, in which the bottom layer was a switchable VO_2_ radiative cooler and the top layer was a spectrally selective filter. The top layer, composed of Ge/MgF_2_ with low transmissivity in the solar spectrum, served as a sunshade, preventing solar irradiation from reaching the bottom radiative cooler owing to strong absorption by Ge in the solar spectral range. Furthermore, the top layer acted as a band pass filter with high transmissivity in the atmospheric window, thereby suppressing the emissivity of the bottom layer in the remainder of the IR regime with low transmissivity. These self-adaptive RCMs can provide RC when the temperature is above T_c_ and automatically turn off RC when the temperature is below the T_c_. In the “on” state, these self-adaptive RCMs exhibit high thermal emissivity across the atmospheric window, while minimizing absorption in the other wavelength ranges. By contrast, when the temperature is below T_c_, these self-adaptive RCMs exhibit minimum thermal emissivity across the entire thermal wavelength range to turn off the RC capability.

Enhanced thermal energy management of RCMs by using energy-storage PCMs has been reported. Paraffin-based PCMs store and release thermal energy with high thermal capacity during isothermal phase transitions, and they have been widely utilized in thermal energy storage and management [112, 113]. A bifunctional paint consisting of SiO_2_-PCM core–shell NPs and MPs was reported for thermal energy storage and efficient passive cooling (Fig. 7c and d) [114]. The SiO_2_-PCM was synthesized by means of surface polymerization and dispersal in alcohol, and the bifunctional paint was prepared by mixing the prepared SiO_2_-PCM with acrylic resin, which is a radiative polymeric binder. The bifunctional paint exhibited a high solar reflectivity of 95.6% owing to the presence of MPs of diameters ranging from 0.5 to 1.5 μm, which triggered a strong Mie-scattering effect to reflect sunlight. Meanwhile, SiO_2_ with strong phonon-polariton resonance at 9.7 μm induced a high emissivity of 95.95% in the 8–13 μm wavelength region. Compared to SiO_2_ paint without PCMs, in outdoor measurements, the bifunctional paint exhibited enhanced cooling performance in terms of temperature drop and the time-buffering effect, which was attributed to its latent heat storage capacity during the isothermal phase transition of the PCM (Fig. 7e).

## Multifunctional RCMs

Multifunctional RCMs that employ a combination of functional NPs have been reported. Lee et al. presented a self-classifying smart device that has a display, an ultraviolet sensor, and RC functions [115]. This device has three functions as follows: (1) it emits green light in response to an alternating current, (2) it emits green light upon exposure to UV light, (3) it exhibits the RC property. This device consisted of a PDMS composite layer in which NPs and giant core–shell QDs were dispersed between an indium tin oxide (ITO) electrode and an Ag electrode. An Ag thin film was used as the bottom electrode, and ITO-coated polyethylene terephthalate (ITO PET) was used as the top electrode to maximize the reflection of visible light. The active PDMS layer is an excellent mid-IR emitter and transparent visible medium. Cu-doped ZnS NPs dispersed in a dielectric layer act as electroluminescence (EL) phosphors, emitting blue light in response to alternating current (AC) in a display. CdSe/ZnS QDs were designed with a green-light-emitting CdSe core and a UV-light-absorbing ZnS shell structure. These CdSe/ZnS QDs were band-engineered to emit green light at 527 nm, absorb UV light, and maximize the Stokes shift to emit visible light. Upon the application of AC, only the Cu-doped ZnS selectively emits 450 nm of blue electroluminescent light. By contrast, the QDs emit green PL at 527 nm under irradiation with 365 nm UV light in the absence of applied AC. In outdoor measurements, the proposed smart device was cooler by 9.6 ℃ during the daytime. Compared to the RC device without Cu-doped ZnS NPs and CdSe/ZnS QDs, the cooling performance of the smart device was weaker owing to solar energy absorption by the Cu-doped ZnS and GCs. In the nighttime, both RC devices were cooler by 8.6 ℃ because no solar energy was absorbed by the ZnS and GCs. This self-classifying smart device was able to successfully classify the input signals of AC and UV light and provide a spectrum-selective response. Furthermore, this multifunctional RC device finally embodied excellent RC performance in an outdoor environment by emitting green or blue light. Jeon et al. demonstrated multifunctional light-emitting PDRC materials by embedding light-emitting materials into a radiative polymer layer. [116]. These RCMs did not exhibit any color in the visible spectrum, but they emitted bright luminescence under UV light or blue illumination (Fig. 7f). Silica-coated perovskite NPs were selected as the light-emitting materials owing to their superior optical properties such as high photoluminescence quantum yield, high phonon conversion efficiency, and high chemical stability. In addition, these perovskite NPs produce minimum amounts of phonons under sunlight and generate adequate photons, which makes them highly suitable for use as light-emitting materials. Given that the molecular vibration bonds of PDMS absorb and emit thermal energy in the 8–13 μm wavelength range, PDMS was utilized to build the IR emissive matrix of RCMs. In this design, the RCMs simultaneously exhibited passive radiative cooling and light-emitting properties. The emission colors were tailored by modifying the composition of perovskite NPs (CsPbBr_3_: green, CsPbBr_x_I_3-x_: red). The SiO_2_-perovskite-NP-based RC materials exhibited low absorptivity in the 0.3–2.5 μm wavelength range (< 0.1) and high emissivity in the 8–13 μm wavelength range (> 0.9). Moreover, they achieved sub-ambient temperature drops of 6.6, 7.4, and 6.0 ℃ for PDMS only, PDMS with CsPbBr_3_, and PDMS with red CsPbBr_x_I_3-x_ QDs, respectively, during the daytime (Fig. 7g). Furthermore, smart house systems, which change their apparent color under white light and UV light, were proposed to demonstrate the potential applications of RC materials such as signboards, advertisements, patterns for components of smart buildings, or even anticounterfeiting systems (Fig. 7h).

## Conclusion

In sum, as the demand for cooling-related energy supply increases, RC have been emerged as a promising strategy to reduce this energy consumption and mitigate the associated environmental issues. The required properties of PDRC materials are high reflectivity in the solar spectrum and high emissivity within the atmospheric window to dissipate thermal energy into outer space. Colloidal NP- and MP-based RCMs are promising materials for efficient PDRC owing to their tunable properties and facile processing. By adjusting the size, shape, and composition of NPs and MPs, the properties of RCMs can be engineered to achieve high reflectivity in the solar spectrum and high emissivity within the atmospheric window. Table 1 summarizes recent studies on RCMs that utilize colloidal NPs and MPs, which have either been property-engineered for PDRCs or are commercially available. Many ceramic- and oxide-based inorganic NPs and MPs, including SiO_2_, TiO_2_, Y_2_O_3_, ZnO, Al_2_O_3_, CaCO_3_, and BaSO_4_, are stable and chemically inert materials (Table 1) and have been utilized as additives in commercial paints. These materials generally have good heat resistivity, mechanical stability, and chemical resistivity, and their weather resistance and chemical tolerance have been demonstrated in long-term outdoor measurements. However, structure-engineered NPs could be affected by stability issues owing to their large surface area and chemical instability of compounds, which should be considered when utilizing NP-based RCMs in residential and commercial applications. The weak correlation between temperature drop and cooling power density may be attributed to the fact that the temperature drops of RCMs depend strongly on the local atmospheric conditions during outdoor temperature measurements, and the experimental setups and atmospheric conditions differ across studies. Meanwhile, most particle-based RCMs were composed of commercially available inorganic particles, but a few were composed of laboratory-synthesized colloidal particles. To synthesize the colloidal particles for use in RCMs, the sol–gel and hydrothermal methods were utilized because of their simplicity in experimental settings and the fact that their reactions generally proceed in a solution (Additional file 1: Table S1). Although the commercially available particles exhibit promising RC performances and are relatively inexpensive, readily available, and capable of being used in large quantities, they have certain limitations in terms of efficient cooling, for example, UV absorption, weak solar reflectivity, narrow absorption coverage in the atmospheric transparent window, and strong IR absorption outside the atmospheric window. Structure-engineered NPs and MPs allow RCMs to have spectrally selective IR emissivity and high solar reflectivity. Therefore, engineered NPs and MPs, particularly for PDRC, should be studied thoroughly to maximize sub-ambient cooling during the daytime. In addition, most RCMs provide only the cooling functionality. Owing to the large structural diversity of colloidal NPs and MPs, various functionalities can be implemented in RCMs to obtain multifunctional RCMs with enhanced thermal management capabilities. Thus far, inorganic NPs and MPs have been used to provide novel properties such as phonon–polariton resonance, plasmonics, luminescent wavelength conversion, structural colors, and phase change property in RCMs. Smart and multifunctional RCMs are expected to be useful for realizing diverse emerging applications, including energy-saving buildings, vehicle cooling, solar cells, smart fabrics for humans, and smart devices. Therefore, it is important to design novel PDRC devices by assembling combinations of various functional NPs and MPs and to propose new concepts by using property-engineered functional colloids.

**Table 1:** Summary of materials, and properties of RC materials

Materials	Size	Shape	Rsolar	ε	Measurement date	Location	Cooling performance	Refs.
Al_2_O_3_	10–20 nm	Spherical	0.9465	0.9163	May 2019	Seoul, South Korea	Temperature drops of 10.35 ℃ with a cooling power density of 106.43 W/m^2^	[65]
BaSO_4_	400 nm	Spherical	0.976	0.96	March 2018	Indiana, United States	Temperature drops of 4.5 ℃ with a cooling power density of 117 W/m^2^	[51]
BaSO_4_	400 nm	Spherical	0.986	0.981	September 2021	Wuhan, China	Temperature drops of 2.5 ℃ with a cooling power density of 125.8 W/m^2^	[117]
CaCO_3_	1.9 μm (length), 500 nm (diameter)	Rod	0.955	0.94	March 2018	Indiana, United States	Temperature drops of 1.7 ℃ with a cooling power density of 37 W/m^2^	[54]
CaCO_3_	20–30 μm	N/A	N/A	0.896	April 2022	Seoul, South Korea	Temperature drops of 6.52 ℃ with a cooling power density of 93.1 W/m^2^	[118]
MgHPO_4_·1.2H_2_O	100–200 nm	Irregular sheet	0.922	0.94	November 2019	Guangzhou, China	Temperature drops of 4.1 ℃ with a cooling power density of 78.18 W/m^2^	[119]
polysilsequioxane	1.14 µm	Spherical	0.9383	0.9429	May 2022	Hangzhou, China	Temperature drops of 3.6 ℃ with a cooling power density of 56.5 W/m^2^	[120]
SiO_2_	200–450 nm	Spherical	0.7	0.95	N/A	Shenzhen, China	Temperature drops of 11.2 ℃ with a cooling power density of 45 W/m^2^	[60]
SiO_2_	300–700 nm	Spherical	0.97	0.94	N/A	Shenzhen, China	Temperature drops of 15.9 ℃	[61]
SiO_2_	< 1 μm	Spherical	N/A	N/A	N/A	Shanghai, China	Temperature drops of 1.0–2.5 ℃	[121]
SiO_2_	< 5 μm	Spherical	0.96	0.95	October	Nanjing, China	Temperature drops of 6.2 ℃	[122]
SiO_2_	400 nm	Spherical	0.972	0.943	September 2020	Seoul, South Korea	Temperature drops of 6.12 ℃ with a cooling power density of 40.75 W/m^2^	[74]
SiO_2_	8 μm	Spherical	0.96	0.93	October 2016	Arizona, United States	Cooling power density of 93 W/m^2^	[44]
SiO_2_	2 μm	Spherical	0.97	0.94	May	New Mexico, United States	Temperature drops of 12 ℃	[56]
SiO_2_	6–14 μm	Spherical	0.97	0.96	May 2019	Shanghai, China	Temperature drops of 6 ℃ with a cooling power density of 61 W/m^2^	[59]
SiO_2_	10 μm	Spherical	0.96	0.9	N/A	Illinois, United States	Temperature drops of 6.1 ℃ with a cooling power density of 85 W/m^2^	[62]
SiO_2_, SiC	50 nm	Spherical	N/A	0.95	N/A	Sydney, Australia	Temperature drops of 17 ℃ (nighttime)	[64]
SiO_2_, SiC, TiO_2_	50 nm, 50 nm, 1 μm	Spherical	0.907	0.90	September 2016	Shanghai, China	Temperature drops of 5 ℃ with a cooling power density of 150 W/m^2^ (nighttime)	[68]
SiO_2_, BaSO_4_	4 µm, 400 nm	Spherical	0.95	0.96	October 2020	Weihai, China	Temperature drops of 8.1 ℃ with a cooling power density of 89.6 W/m^2^	[123]
SiO_2_, Al_2_O_3_	0.8–1.6 μm	Spherical	0.941	0.935	October 2020	Seoul, South Korea	Temperature drops of 7.9 ℃ with a cooling power density of 100 W/m^2^	[52]
SiO_2_, Al_2_O_3_	40–60 nm, 20–40 nm	Spherical	N/A	0.74	October 2021	Seoul, South Korea	Temperature drops of 9.67 ℃ with a cooling power density of 89.51 W/m^2^	[16]
SiO_2_- Zn_2_SiO_4_ core-shell	1–10 μm	Spherical	0.96	0.94	May 2021	Nanjing, China	Temperature drops of 4.1 ℃ with a cooling power density of 85.8 W/m^2^	[58]
SiO_2_-TiO_2_ core-shell	12.85 μm	Spherical	0.97	0.95	August 2021	Beijing, China	Temperature drops of 5.26 ℃	[124]
TiO_2_	400 nm	Spherical	0.9	0.9	N/A	N/A	Cooling power density of 100 W/m^2^	[69]
TiO_2_, glass MPs	400 nm, 40 μm	Spherical	0.898	0.96	October 2018	Beijing, China	Temperature drops of 6 ℃ with a cooling power density of 84.2 W/m^2^	[57]
TiO_2_, Y_2_O_3_	200–400 nm, 1–5 μm	N/A	0.922	0.949	April 2022	Tianjin, China	Temperature drops of 7.7 ℃ with a cooling power density of 72.5 W/m^2^	[71]
ZnO	300–800 nm	Spherical	0.9	N/A	N/A	California, United States	Temperature drops of 10 ℃ with a cooling power density of 200 W/m^2^	[50]
ZnO	50 nm	N/A	0.97	0.95	May 2021	Nanjing, China	Temperature drops of 4.3 ℃ with a cooling power density of 97.4 W/m^2^	[125]

Solar reflectance (R_solar_) and emissivity (ε) denote the solar reflectivity and IR emissivity of the listed RC materials, respectively

## Supplementary Information

**Additional file 1: Table S1.** Summary of materials, synthetic mechanism, and properties of RC materials. Solar reflectance (R_solar_) and emissivity (ε) denote the solar reflectivity and IR emissivity of the listed RC materials, respectively.

## Data Availability

Not applicable.
