# Supplementary material for: Colloidal inorganic nano- and microparticles for passive daytime radiative cooling
Source: Nano Converg. 2023 Apr 18;10:17. doi: 10.1186/s40580-023-00365-7 (PMC10113424; doi:10.1186/s40580-023-00365-7)
Supplement: Supplementary file 1 — Additional file 1: Table S1. Summary of materials, synthetic mechanism, and properties of RC materials. Solar reflectance (Rsolar) and emissivity (ε) denote the solar reflectivity and IR emissivity of the listed RC materials, respectively. [file 40580_2023_365_MOESM1_ESM.docx]

Supporting Information:

**Colloidal Inorganic Nano- and Microparticles for Passive Daytime Radiative Cooling**

Ho Young Woo, Yoonjoo Choi, Hyesun Chung, Da Won Lee, Taejong Paik*

School of Integrative Engineering, Chung-Ang University, Seoul 06974, Republic of Korea

* Corresponding author: Taejong Paik

Tel.: +82-2-820-5435

E-mail address: paiktae@cau.ac.kr

**Table S1** Summary of materials, synthetic mechanism, and properties of RC materials. Solar reflectance (R_solar_) and emissivity (ε) denote the solar reflectivity and IR emissivity of the listed RC materials, respectively.

| **Materials** | **Synthetic mechanism** | **Size** | **Shape** | **R_solar_** | **ε** | **Cooling performance** | **Ref.** |
| --- | --- | --- | --- | --- | --- | --- | --- |
| SiO_2_-Zn_2_SiO_4_ core-shell | Sintering | 1–10 μm | Spherical | 0.96 | 0.94 | Temperature drops of 4.1 ℃ with a cooling power density of 85.8 W/m^2^ | [1] |
| SiO_2_-TiO_2_ core-shell | Sol-gel | 12.85 μm | Spherical | 0.97 | 0.95 | Temperature drops of 5.26 ℃ | [2] |
| MgHPO_4_·1.2H_2_O | Hydrothermal synthesis | 100–200 nm | Irregular sheet | 0.922 | 0.94 | Temperature drops of 4.1 ℃ with a cooling power density of 78.18 W/m^2^ | [3] |
| polysilsequioxane | Sol-gel | 1.14 µm | Spherical | 0.9383 | 0.9429 | Temperature drops of 3.6 ℃ with a cooling power density of 56.5 W/m^2^ | [4] |
| SiO_2_ | Sol-gel | 200–450 nm | Spherical | 0.7 | 0.95 | Temperature drops of 11.2 ℃ with a cooling power density of 45 W/m^2^ | [5] |
| SiO_2_ | Sol-gel | 300–700 nm | Spherical | 0.97 | 0.94 | Temperature drops of 15.9 ℃ | [6] |
| SiO_2_ | Sol-gel | < 1 μm | Spherical | N/A | N/A | Temperature drops of 1.0–2.5 ℃ | [7] |
| SiO_2_ | Sol-gel | < 5 μm | Spherical | 0.96 | 0.95 | Temperature drops of 6.2 ℃ | [8] |
| SiO_2_ | Sol-gel | 400 nm | Spherical | 0.972 | 0.943 | Temperature drops of 6.12 ℃ with a cooling power density of 40.75 W/m^2^ | [9] |

**References**

1. J. Huang, M. Li, D. Fan, Appl. Mater. Today **25**, 101209 (2021)

2. D. Hu, S. Sun, P. Du, X. Lu, H. Zhang, Z. Zhang, Compos. Part A Appl. Sci. Manuf. **158**, 106949 (2022)

3. X. Huang, N. Li, J. Wang, D. Liu, J. Xu, Z. Zhang, M. Zhong, ACS Appl. Mater. Interfaces **12**(2), 2252-2258 (2019)

4. T. Zuo, J. Zhang, S. Zhong, T. Xu, L. Xu, S. Xu, B. Pan, Y. Cai, L. Yi, Mater. Today Commun. **32**, 104096 (2022)

5. Y. Zhang, J. Yu, ACS Appl. Nano Mater. **4**(10), 11260-11268 (2021)

6. Y. Zhang, J. Yu, ACS Appl. Mater. Interfaces **14**(40), 45707-45715 (2022)

7. R. Xiao, C. Hou, W. Yang, Y. Su, Y. Li, Q. Zhang, P. Gao, H. Wang, ACS Appl. Mater. Interfaces **11**(47), 44673-44681 (2019)

8. B. Xiang, R. Zhang, Y. Luo, S. Zhang, L. Xu, H. Min, S. Tang, X. Meng, Nano Energy **81**, 105600 (2021)

9. T. Y. Yoon, S. Son, S. Min, D. Chae, H. Y. Woo, J.-Y. Chae, H. Lim, J. Shin, T. Paik, H. Lee, Mater. Today Phys. **21**, 100510 (2021)
